# Catalyst Electrodes with PtCu Nanowire Arrays In Situ Grown on Gas Diffusion Layers for Direct Formic Acid Fuel Cells

**DOI:** 10.1021/acsami.1c24010

**Published:** 2022-02-24

**Authors:** Yang Li, Yichang Yan, Yanping He, Shangfeng Du

**Affiliations:** † School of Chemical Engineering, University of Birmingham, Birmingham B15 2TT, U.K.; ‡ School of Chemical Engineering, Kunming University of Science and Technology, Kunming 650504, China

**Keywords:** direct formic acid fuel cells (DFAFCs), gas diffusion electrode (GDE), PtCu, nanowire, formic acid oxidation

## Abstract

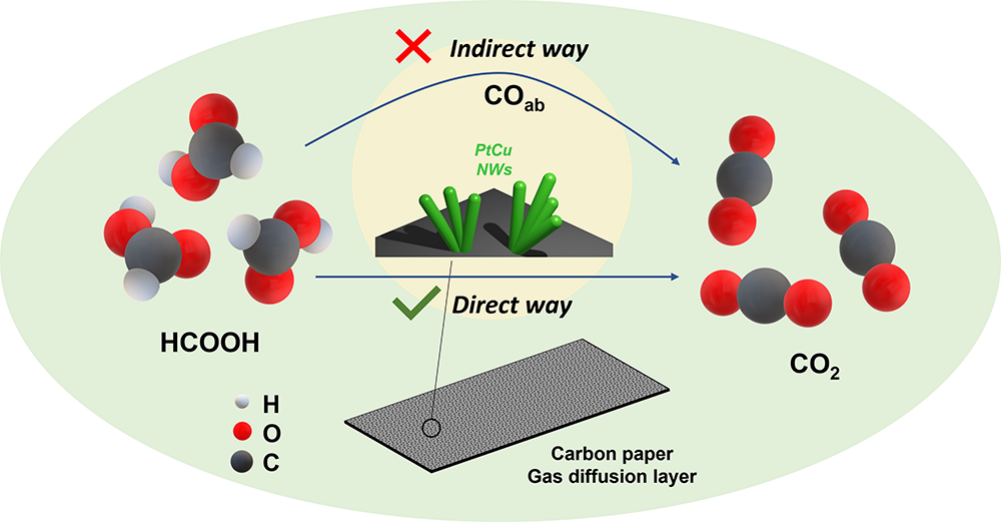

The excellent performance and safety of direct formic acid fuel cells (DFAFCs) promote them as potential power sources for portable electronic devices. However, their real application is still highly challenging due to the poor power performance and high complexity in the fabrication of catalyst electrodes. In this work, we demonstrate a new gas diffusion electrode (GDE) with ultrathin PtCu alloy nanowire (NW) arrays in situ grown on the carbon paper gas diffusion layer surface. The growing process is achieved by a facile template- and surfactant-free self-growth assisted reduction method at room temperature. A finely controlled ion reduction process tunes the nucleation and crystal growth of Pt and Cu leading to the formation of alloy nanowires with an average diameter of about 4 nm. The GDE is directly used as the anode for DFAFCs. The results in the half-cell GDE measurement indicate that the introduction of Cu in PtCu NWs boosts the direct oxidation pathway for formic acid. The Pt_3_Cu_1_ NW GDE shows a 2.4-fold higher power density compared to the Pt NW GDE in the membrane electrode assembly test in single cells.

## 1 Introduction

Over the last decades, the pace of research and development of clean and sustainable energy has sharply increased, motivated by the growing energy demand and pressures of environmental challenges. Fuel cells, as one of the clean power generation technologies, can directly convert the chemical energy of fuel into electrical power with low CO_*x*_ and NO_*x*_ emissions^1^ and thus have become a crucial industrial sector for global sustainable economic development. Among various type of fuel cells, the proton exchange membrane fuel cell (PEMFC) is receiving ever-increasing attention because of its low operating temperature and high energy efficiency.^2,3^ In the development of PEMFCs, most efforts were focused on hydrogen PEMFCs and direct methanol fuel cells (DMFCs). However, some of their inherent limitations have still not been addressed even with such intensive studies. For example, hydrogen storage is a critical part for the hydrogen PEMFC, but the high cost and low storage weight ratio are still challenging. Regarding the DMFC, although a number of studies have already been reported in reducing the methanol crossover,^4−6^ it is still the most significant limiting factor for improving the fuel utilization and cell power performance. To overcome these limits of the hydrogen PEMFC and DMFC, the development of direct formic acid fuel cells (DFAFCs) attracts increasing efforts in recent years.

With the DFAFC development, the sluggish formic acid oxidation (FAO) and catalyst poisoning caused by the reaction intermediate species (i.e., CO) are the major challenges. In order to address both issues, the strategy of alloying Pt with a second metal to form Pt-based alloy catalysts has been proposed. A number of attempts of Pt–M catalysts (M = Au, Ag, Bi, Zn, Cu, Co, and Pd) have been demonstrated, showing improved catalytic activities toward the FAO.^7−12^ Such enhancement was believed to result from the downshift of the d-band center of Pt due to the additional metal and, most importantly, the increased tolerance against adsorbed intermediates. For example, Luo et al. synthesized a novel PtSnBi intermetallic catalyst, which suppressed the formation of CO intermediates and optimized dehydrogenation steps.^13^ The mass activity of the catalyst reached 4.39 A mg^–1^, more than 40 times higher than that of a commercial Pt catalyst. In addition to the control of composition, structure engineering has been another way to improve electrocatalytic performance. Among various structures, one-dimensional (1D) nanostructures, such as nanowires, nanorods, nanotubes, nanochains, etc.,^14−19^ have received increasing prominence in recent studies. Compared with nanoparticles, 1D nanostructures show good potential to alleviate the inherent drawback resulting from aggregation, dissolution, and Ostwald ripening.^20^ In addition, it has been well demonstrated that 1D nanostructures can facilitate electrocatalytic activity via exposing highly active crystal facets, along with promoting electron transport through the path directing effect.^21^

However, most studies on the catalyst toward the formic acid oxidation only focused on model studies, and their experiments were usually carried out in the evaluation of their intrinsic activities using a half cell electrochemical measurement with a thin-film catalyst electrode in liquid electrolytes. Research studies have demonstrated a big disparity for highly active shape-controlled catalysts between their intrinsic catalytic activities measured in liquid electrolytes and their power performance within practical catalyst electrodes in fuel cell devices.^22^ Most of these catalysts showed limited improvement or even worse power performance compared to commercial catalysts in the single fuel cell test, compared with their excellent catalytic activities revealed in the half cell electrochemical measurement, which is mainly caused by the mass transport resistance under the complex environment conditions within the practical electrodes during the fuel cell operation.^8,9,23^ Therefore, to fabricate practical electrodes based on these shape-controlled catalysts, a new electrode approach is urgently required to improve mass transport resistance thus achieving high power performance. Here, we demonstrate a facile method to fabricate gas diffusion electrodes (GDEs) by directly growing PtCu nanowire (NW) arrays on the carbon paper gas diffusion layer (GDL) surface at room temperature without using any template or surfactant. The PtCu NW GDE shows great performance in both the half-cell GDE measurement and membrane electrode assembly (MEA) test in single cells due to advantages of significantly reduced mass transport resistance of the nanowire array catalyst layer and enhanced direct oxidation pathway for formic acid by introducing Cu atoms. Comparing to traditional wet chemical methods (such as sodium borohydride or ethylene glycol reduction) where catalysts are first formed on the carbon support, separated, and dried to prepare the catalyst black, then dispersed to make catalyst ink, and finally coated onto GDLs to fabricate GDEs, the method demonstrated here can prepare the GDE in a one-step process from the metal precursor and GDL and can be directly employed to assemble DFAFCs.

## 2 Experiment

### 2.1 In Situ Growing PtCu Nanowire Arrays on GDLs

**Figure 1:**
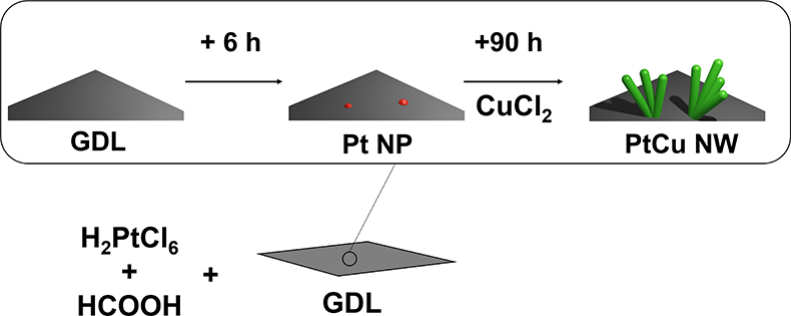
Scheme illustration for the growth of PtCu nanowire (NW) arrays on the GDL surface to fabricate a PtCu NW GDE.

PtCu NW GDEs were fabricated with the formic acid reduction method reported with our group before with some changes.^14^ Typically, to grow PtCu NWs with a metal loading of 2.0 mg_PtCu_ cm^–2^ on the GDL surface, a mixed solution of H_2_O, HCOOH, and H_2_PtCl_6_ was added into a Petri-dish followed by the introduction of a piece of GDL. The sample was left at room temperature for 6 h before adding CuCl_2_ solution. After another 90 h, the color of the solution changed to colorless, and the reaction was completed. The GDL with PtCu NW arrays was finally washed with IPA and H_2_O followed by drying in the oven at 40 °C. The fabrication process is illustrated below in Figure 1. To synthesize catalysts with various PtCu metal ratios, the varied amount of Pt and Cu precursors were applied with the constant metal loading, and they are labeled as Pt NW GDE (2.0 mg_Pt_ cm^–2^), Pt_5_Cu_1_ NW GDE (1.88 mg_Pt_ cm^–2^ + 0.12 mg_Cu_ cm^–2^), Pt_3_Cu_1_ NW GDE (1.80 mg_Pt_ cm^–2^ + 0.20 mg_Cu_ cm^–2^), and Pt_1_Cu_1_ NW GDE (1.50 mg_Pt_ cm^–2^ + 0.50 mg_Cu_ cm^–2^). The prepared GDL with catalyst nanowire arrays were directly used as the GDE for the following MEA test.

### 2.2 Physical Characterization

The surface morphologies of the PtCu NW GDE were characterized using a scanning electron microscope (SEM) (Jeol 7000F). Transmission electron microscopy (TEM) samples were prepared using catalysts scraped from the GDE surface and dispersed on a Au grid (300 mesh), and the analysis was conducted using a Jeol 2100 TEM. X-ray diffraction (XRD) patterns were recorded with a Bruker D8 Autosampler using Cu Κα radiation (λ = 0.15418 nm) between 2θ values of 20 and 80°. The chemical valences of Pt and Cu in the GDE were analyzed using X-ray photoelectron spectroscopy (XPS) (Thermo Fisher Scientific NEXSA spectrometer with a 72 W micro-focused monochromatic Al Κα source), and data analysis was conducted using CasaXPS software with correction of the C 1s peak at 284.8 eV as a reference. The element content of the catalysts was analyzed using inductively coupled plasma mass spectrometry analysis (ICP-MS, Perkin Elmer Nexion 300X, USA) with a plasma strength of 1500 W.

### 2.3 Half-Cell GDE Measurement

The half-cell GDE measurement was conducted using a FlexCell polytetrafluoroethylene (PTFE) from Gaskatel, which was developed in our previous studies.^24^ The principle of the half-cell GDE measurement is based on a three-electrode system. Prior to measurement, the diluted Nafion solution was coated onto the surface of the GDE. A commercial HydroFlex RHE was employed, while a built-in platinum coil was used as the counter electrode. The GDE was initially measured in 1.0 M HClO_4_ aqueous solution and purged with N_2_ gas for 30 min to remove O_2_ before testing. The working electrode was electrochemically cleaned by 100 cycles of cyclic voltammetry (CV) scan in the range of 0.05–1.2 V vs RHE with a scan rate of 100 mV s^–1^ followed by running three CV cycles in the range of 0.05–1.2 V vs RHE at a scan rate of 20 mV s^–1^, and the last cycle was used for analysis. Then, the electrolyte was changed to 1.0 M HClO_4_ containing 1.0 M formic acid. It was then purged with N_2_ gas for 30 min followed by recording of the CV between 0.2 and 1.2 V with a scan rate of 20 mV s^–1^.

### 2.4 Membrane Electrode Assembly Test

The as-prepared GDE was used as the anode, and a commercial GDE (catalyst loading: 2.0 mg_Pt_ cm^–2^) was used as the cathode for fabricating MEAs. An IPA solution of Nafion DE 1021 (volume ratio of DE 1021 to IPA = 1:2) was sonicated using a sonic bath for 5 min and then was painted onto the GDE surface (Nafion loading at 1.2 mg cm^–2^) and dried under an infrared lamp for 2 h. The MEA with an active area of 4 cm^2^ was fabricated by hot pressing the anode, cathode, and Nafion 212 membrane at 135 °C under a pressure of 4.9 MPa for 2 min. The MEA test was performed at 75 °C with an 850e Multi-Range Fuel Cell Test System (Scribner Associates Inc., US). Two Teflon films with a thickness of 254 μm were used as the gasket at both sides. The anode was fed with 3 mol L^–1^ formic acid at a flow rate of 1 mL min^–1^, while the cathode was fed with dry air at a flow rate of 300 mL min^–1^ without backpressure. Then, polarization curves were recorded at a scan rate of 2 mV s^–1^. To evaluate the stability of the GDE in DFAFCs, an accelerated degradation test (ADT) of the anode was conducted. For this test, the anode was fed with deionized water with a flow rate of 1 mL min^–1^ and the cathode was fed with dry hydrogen at 300 mL min^–1^ serving as both counter and reference electrode, also designated as a dynamic hydrogen electrode (DHE).^25^ The potential scan between 0.05 and 1.2 V vs DHE with a scan rate of 100 mV s^–1^ was performed for 3000 cycles, and CV was recorded at the 1st, 1000th, 2000th, and 3000th cycle with a scan rate of 20 mV s^–1^.

## 3 Results and Discussion

### 3.1 Physical Characterization

**Figure 2:**
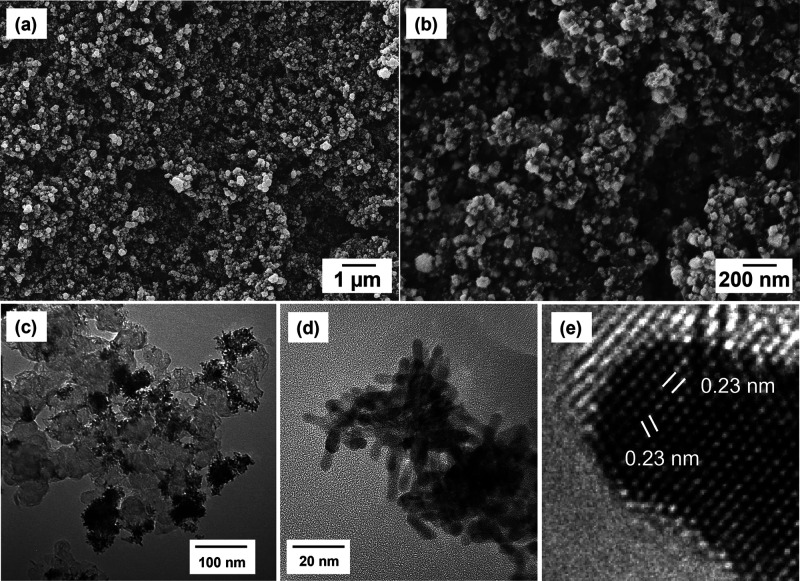
(a, b) SEM images of the Pt_3_Cu_1_ NW GDE with different magnifications, (c, d) TEM images of Pt_3_Cu_1_ NWs scraped from the GDE with different magnifications, and (e) a high-resolution TEM image showing the interlattice spacing of a Pt_3_Cu_1_ nanowire.

In order to fabricate PtCu NW electrodes that can be directly employed in the DFAFC, PtCu NW arrays were grown on the GDL surface in an aqueous solution using chloroplatinic acid and copper(II) chloride as the metal precursors. The SEM images and EDX element map of the Pt_3_Cu_1_ NW GDE (Figure S1) indicate that both Pt and Cu are uniformly reduced on the GDL surface. This indicates that using the formic acid reduction method can successfully form PtCu on the GDL surface. To understand distribution and structure of the PtCu NW, SEM and TEM analysis were conducted to the Pt_3_Cu_1_ NW GDE surface and the catalyst scrapped from the electrode, and images are shown in Figure 2. It can be seen that Pt_3_Cu_1_ NWs are uniformly grown on the GDL surface. They cover the surface of carbon spheres, which are part of the microporous layer of the GDL, stretching out to form a nanowire array cluster with a size of about 50–100 nm. The TEM image shows that these nanowires have an average length of around 20 nm with a diameter of about 4 nm. The lattice fringe patterns (Figure 2e) indicate the single crystal feature and an average interlattice spacing of 0.23 nm, confirming the anisotropic growth of the single crystal nanowire along the Pt <111> direction.

The influence of the Cu content was also investigated, and surface SEM images of the PtCu nanostructures with different ratios of Pt to Cu on the GDL are shown in Figure S2. Compared to the relatively uniform distribution of Pt NWs on the GDL surface where only a few obvious clusters are shown in the image, the introduction of Cu results in the formation of large particle-like clusters and this becomes severer with the increasing Cu content. A closer inspection (Figure S3) reveals the detailed structures of these catalyst clusters. The typical cluster size of the PtCu NW catalyst is larger than 50 nm and some reach around 100 nm. The Pt NW catalyst, on the other hand, has a much smaller cluster size (10–20 nm), which is the reason why it shows a relatively clean surface on the SEM image as plenty of small clusters are below the analysis resolution (Figure S2a). To determine the metal ratio of the synthesized catalysts, ICP-MS analysis was performed to the as-prepared PtCu GDEs and the results are summarized in Table S1. Good consistency is demonstrated with the expected Pt:Cu ratios.

The growth mechanism of the PtCu nanowire using this formic acid reduction method can be attributed to the different standard reduction potentials of Pt and Cu ions during the reaction. From the viewpoint of thermodynamics, the potential of formic acid is +0.25 V vs SHE, which is lower than +0.70 V vs SHE of [PtCl_6_]^2–^ and + 0.34 V vs SHE of Cu^2+^, providing the opportunity for formic acid as a reducing agent to reduce both precursors. However, the very close value with the Cu ion makes its reduction extremely difficult at room temperature in the real application. This was demonstrated with our additional experiments. With formic acid in the absence of a Pt precursor, the color of the CuCl_2_ solution did not change after 96 h and no Cu metal was detected on the GDL surface. When the Pt ion is present in the reaction solution, it can facilitate the reduction of the Cu ion to form PtCu nanowires on the GDL surface. A similar synergy effect during the reduction process has also been reported for Pt and Ni ions.^26^ On the other hand, from the viewpoint of kinetics, the growth mechanism of nanowires might be contributed by two aspects: First, the slow reduction rate provides the opportunity for anisotropic growth.^27^ The order of facet energy is (111) < (100) < (110) for the Pt fcc structure, which facilitates the growth along with the closed-packed <111> direction following the lowest-energy principle. In addition, during the formic acid reduction process, the working reducing agent is a formate anion that is produced from formic acid via the dehydration reaction. At the same time, CO can also be formed as the intermediate species during the process, which will be firmly adsorbed onto the catalyst surface, inhibiting its further growth. Previous studies reported that the dehydration of formic acid is favored on other Pt crystal facets compared with (111) facets.^15^ This thus assists the growth of Pt along the <111> direction to form a 1D nanostructure. Regarding the role of Cu ions, the adsorption of CO intermediate species on Cu surface is very weak. This leads to the growth of Cu without a preferred direction. So, the shape inducing effect of Pt is necessary for growing PtCu nanowires, and the increased Cu content facilitates a non-directional growth, finally forming the shorter nanowires and bigger clusters (Figure S4).

**Figure 3:**
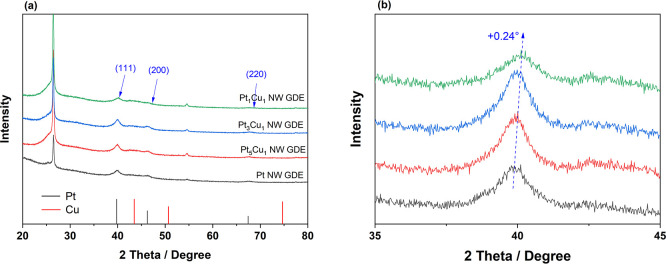
(a) XRD patterns of the Pt NW and PtCu NW GDEs with various Cu contents. (b) Comparison of Pt(111) peak for the Pt NW and different PtCu NW GDEs.

The XRD patterns of the PtCu NW GDEs with various Cu contents are shown in Figure 3. The distinctive peaks at two-theta values of 26.6 and 54.2° are indexed to the (002) and (004) facets of the graphitic carbon of the GDL, respectively, while the rest of the peaks are well indexed to the PtCu alloy. The peaks at 40, 46, and 67° are indexed to the (111), (200), and (220) crystal facets of face-centred cubic (fcc) Pt (JCPDS: 04-0802), respectively. The position of each peak of PtCu is located between the corresponding reference peaks of Pt (JCPDS: 04-0802) and Cu (JCPDS: 04-0836), and no characteristic Cu peak is detected in the XRD patterns. The peaks for PtCu NW show a positive shift, and this increases with the increasing Cu content. Pt_1_Cu_1_ exhibits a shift of 0.24° at two-theta compared to that of the Pt NW. This shift is related to the decrease of the crystal spacing when the small Cu atoms enter the Pt lattice. These pieces of evidence all indicate the successful incorporation of Cu into the Pt lattice structure, and forming a PtCu alloy. In addition, the peak intensity reduces at the high Cu content. This can be ascribed to the lattice contraction caused by the different sizes of Pt and Cu atoms, which decreases the crystallinity of the PtCu NW.

To understand surface composition of the PtCu catalyst, X-ray photoelectron spectroscopy (XPS) was performed on the PtCu NW GDEs with various Cu contents. The peak fitting (Figure S5) of the Pt 4f and Cu 2p spectrum demonstrates that Pt and Cu are both effectively reduced to their metallic state (Pt(0) and Cu(0)), leading to the dominated peaks in each element. This result further shows the effectiveness of the formic acid reduction method to synthesize 1D PtCu catalysts. Table S2 compares the percentage of the metallic state of Pt in different PtCu catalysts. A higher composition of the metallic state is obtained with the increasing Cu content. This increasing trend supports the discussion above about the formation of nanowires, where a synergy effect occurs between both metal ions during the reduction process. Table S2 also reveals a negative shift of binding energy for all three PtCu catalysts compared with the Pt NW, and this increases with the increasing Cu content. Previous studies have demonstrated that the shift of binding energy can lead to change of the d-band center, which is related to the binding energy of the intermediates and the pathway of the formic acid oxidation.^28,29^ The negative shifts of 0.041, 0.049, and 0.15 eV are recorded for the Pt_5_Cu_1_, Pt_3_Cu_1_, and Pt_1_Cu_1_ NW, respectively, suggesting that the introduction of Cu atoms can optimize their electronic structure.

### 3.2 Electrode Performance

**Figure 4:**
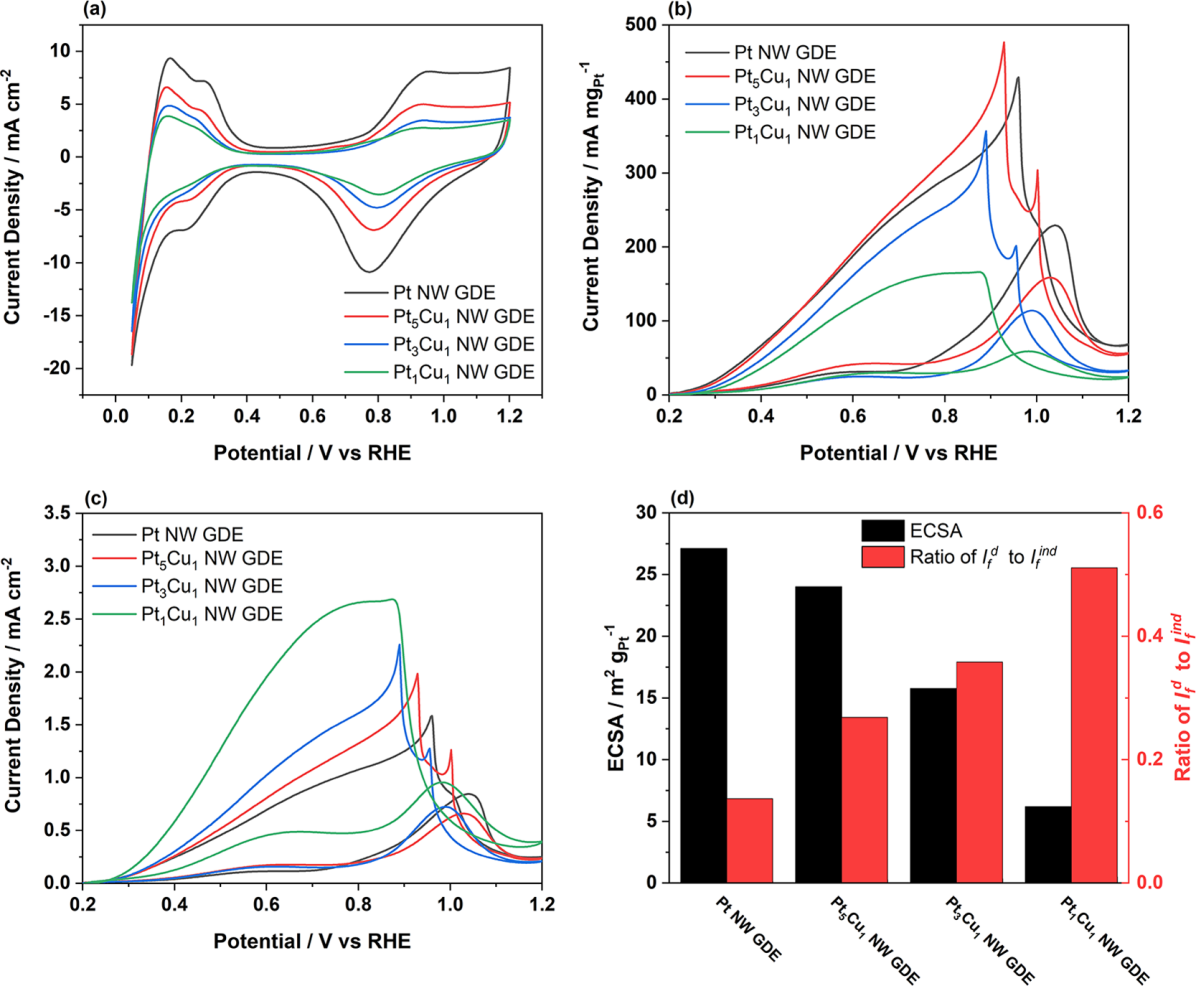
Comparision of CV plots of the Pt NW GDE and the PtCu NW GDEs with different Pt to Cu ratios, recorded using the half-cell GDE measurement in N_2_ saturated (a) 1.0 M HClO_4_, and (b, c) 1.0 M HCOOH +1.0 M HClO_4_ (the current is normalized to the corresponding (b) Pt mass and (c) ECSA, respectively). (d) The trend of ECSA and *I_f_^d^*/*I_f_^ind^* for the Pt NW and PtCu NW GDEs.

The factors that determine the electrode performance are very complex, which not only depends on catalysts themselves but also relies on the environment in operating fuel cells. Therefore, to evaluate the catalytic activity of the as-prepared PtCu NW GDEs toward the formic acid oxidation in a clean environment, electrochemical measurements were performed using the ex situ half-cell GDE measurement. Unlike the thin-film rotating disk electrode (RDE) technique, which is principally unable to predict the power performance of fuel cell catalysts, the half-cell GDE measurement conducted under close conditions to PEMFC operation provides very similar results as those from the MEA test in single fuel cells and offers accurate trends in fuel cell catalytic activity.^30^Figure 4a compares CV plots of the Pt NW and PtCu NW GDEs with different Cu contents recorded in N_2_-purged 1.0 M HClO_4_ aqueous solution at a scan rate of 20 mV s^–1^. It is shown that the hydrogen adsorption/desorption peak of the Pt NW GDE decreases after the introduction of Cu, and the higher Cu content leads to a lower peak. The electrochemical surface areas (ECSAs) of the Pt_5_Cu_1_, Pt_3_Cu_1_, and Pt_1_Cu_1_ NW GDEs are 24.1, 15.8, and 6.2 m^2^ g_Pt_^–1^, respectively, compared to 27.1 m^2^ g_Pt_^–1^ of the Pt NW GDE. One reason for the smaller ECSA is because Cu is not an active metal for hydrogen adsorption/desorption in this situation, and its alloying occupies the active sites on the Pt nanowire surface. Therefore, the ECSA decreases with the increasing Cu content. Another reason is due to the severe agglomeration of PtCu NWs, which can block active sites and reduce the catalyst utilization, thus accelerating the dropping of the ECSA with the increasing Cu content.

With regards to the formic acid oxidation, the most commonly accepted mechanism is a “dual pathway mechanism”.^31^ In one pathway, formic acid is directly oxidized to carbon dioxide via a dehydrogenation reaction without forming any intermediate species:
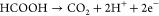
1

In the second pathway, the formic acid oxidation occurs via a dehydration reaction and forms adsorbed intermediates that are mainly carbon monoxide (CO):
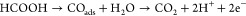
2

These intermediates (mainly CO) can strongly adsorb on the Pt catalyst surface and block active sites leading to catalyst poisoning, which may cause fatal deactivation and poor stability. In order to investigate catalytic activity of the PtCu NW GDEs toward the formic acid oxidation, their CV were recorded in 1.0 M HClO_4_ aqueous solution in the presence of 1.0 M formic acid. As shown in Figure 4b, there are two oxidation peaks with the forward scan for all electrocatalysts, which result from the “dual pathway mechanism” of the formic acid oxidation. The first peak at about 0.6 V is ascribed to the direct formic acid oxidation to CO_2_ (*I_f_^d^*), while the second peak at about 1.0 V is attributed to the oxidation of the adsorbed CO intermediates to CO_2_ (*I_f_^ind^*).^32,33^ The ratio between the current density of the first to the second peak (*I_f_^d^*/*I_f_^ind^*) indicates the tendency of the direct and indirect ways during the formic acid oxidation. A high ratio indicates a more pronounced direct way and less poisoning of the catalytic surface by the CO intermediates. In the case of PtCu catalysts, the ratios are 0.27, 0.39, and 0.51 for the Pt_5_Cu_1_, Pt_3_Cu_1_, and Pt_1_Cu_1_ NW GDEs, respectively. Compared with the ratio of the Pt NW GDE of 0.14, the introduction of Cu atoms significantly increases the ratio between the direct and indirect ways, while the PtCu NW GDE based on the higher Cu content favors the direct pathway more. This enhancement of the direct formic acid oxidation pathway can be attributed to both the (i) optimized electrode structure mentioned above and also the (ii) third-body effect. The third-body effect is based on the fact that the formation of the CO intermediates through the indirect formic acid oxidation cannot happen on isolated Pt atoms because the dehydrate reaction requires at least two adjacent Pt atoms.^32,34^ In the PtCu alloy nanowires, the arrangement of Pt atoms is interrupted by the additional Cu atoms, forming a number of isolated Pt atoms. As a consequence, the direct pathway is enhanced on such isolated Pt atoms, thus enabling a higher ratio. This enhanced direct pathway is also shown in the inspection of ECSA normalized CV curves. As shown in Figure 4c, the peak current density of the Pt_1_Cu_1_ NW GDE is 2.69 mA cm^–2^, which is 1.7 times higher than that of the Pt NW GDE (1.58 mA cm^–2^). In addition, the oxidation peaks in the back scan with all PtCu NW GDEs are observed at <0.86 V vs RHE, which negatively shift compared to the Pt NW GDE, and the shift becomes more significant with the increasing Cu content. This outcome can be ascribed to more hydroxyl groups generated at the presence of Cu atoms, which facilitate the oxidation of the CO intermediates at a low potential.^7,35^ However, although the PtCu NW GDE with the high Cu content shows the enhanced direct pathway and negative shifted oxidation peak, it also exhibits the lowest mass activity (Figure 4b) and ECSA (Figure 4d). This indicates that there are different aspects needed to be considered when the electrocatalyst is chosen for the DFAFC application, and it is necessary to find a balance between their properties.

**Figure 5:**
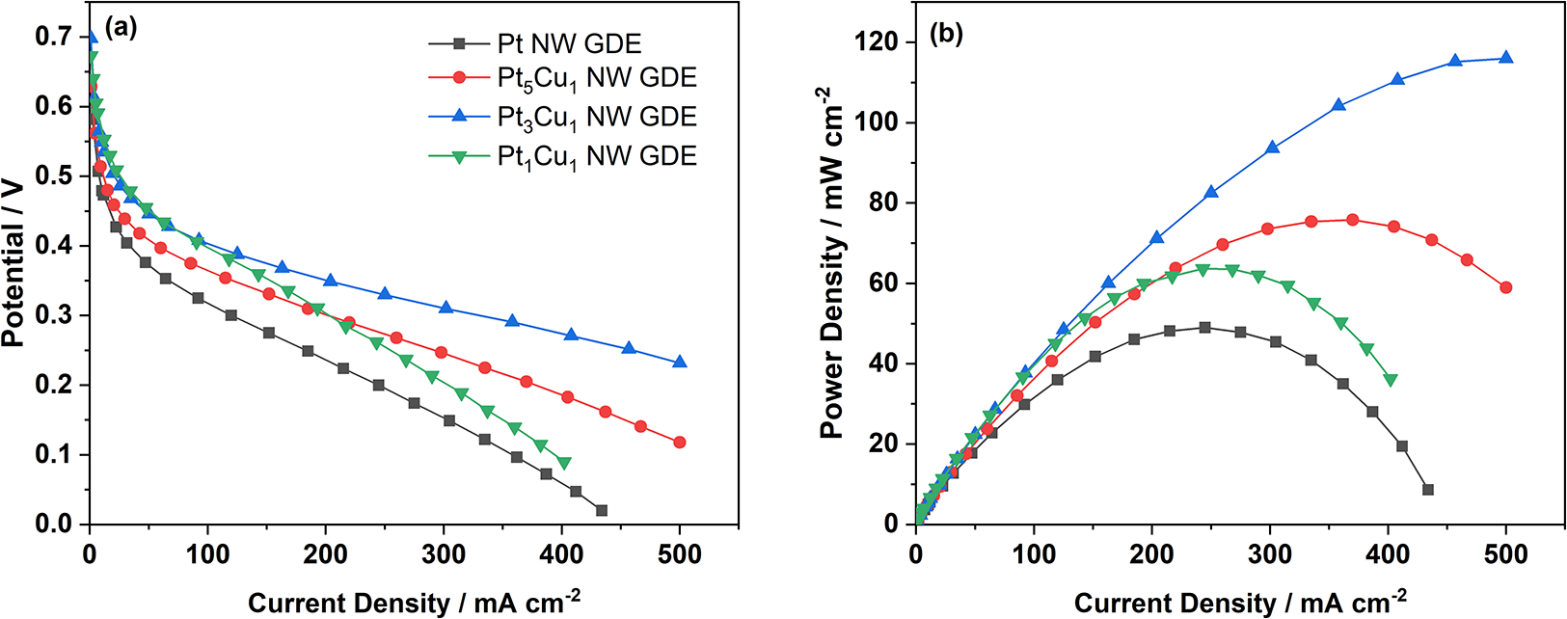
(a) Polarization and (b) power density curves of MEAs with the anode made of the Pt NW GDE and the PtCu NW GDEs with different Cu contents. Test conditions: MEA active area: 4 cm^2^, testing temperature: 75 °C, anode: 3 M formic acid at a flow rate of 1 mL min^–1^, cathode: dry air with a flow rate of 300 mL min^–1^.

Despite the ex situ half-cell GDE test offering trends in the catalytic activity of catalysts in the fuel cell, its operation is still different from the membrane electrode assembly (MEA) test in the single fuel cell device. This is contributed to the complexity of the single-cell system as it is largely dependent on various parameters such as the membrane, ionomer, fuel and product transport, and working environment. As a consequence, the MEA test in the DFAFC single cell is performed to evaluate the real power performance of the as-prepared PtCu NW GDEs. Figure 5 compares the polarization and power density curves of the MEAs with various GDEs.^36^

Polarization curves of the MEAs with the Pt and PtCu NW GDEs are shown in Figure 5a. Within the high potential region, increasing the Cu content leads to a reduced activation loss. This trend can be explained by the dual pathway mechanism of the formic acid oxidation where a lower barrier is requested toward the direct oxidation. The ex situ half-cell GDE measurement has demonstrated that the direct way is promoted at the high Cu content, thus causing a lower voltage loss. Pt_1_Cu_1_, therefore, demonstrates the highest performance in this region. However, its severe agglomeration reduces the ECSA and also increases the mass transfer resistance at the anode, thus leading to a fast drop of the power performance with the increasing current density. Figure 5b compares the power density of the MEAs made from these four GDEs. When increasing the Cu content from Pt to Pt_3_Cu_1_, the peak power density increases by 2.4-fold from 49.1 to 116.3 mW cm^–2^. For Pt_1_Cu_1_, this drops to 63.7 mW cm^–2^. A benchmark GDE made from commercial Pt/C nanoparticles were also fabricated and tested for comparison (Figure S6). Compared to the Pt/C GDE, the PtCu NW GDE shows poorer power performance within the low current density region due to the smaller ECSA resuting from the larger bulk size of the nanowire than that of the Pt catalyst nanoparticle, which has been commonly demonstrated in former research studies.^37^ However, at the medium and large current design regions, a much slower potential drop is shown for the nanowire GDE, which is related to the improved mass transport characteristics within the nanowire catalyst layer compared to the nanoparticle catalyst layer.^21,37^ Within the nanowire electrode, the thinner and porous catalyst layer facilitates transport of both formic acid fuel and produced CO_2_ during the fuel cell operation, in particular at a high catalyst loading, which usually leads to a very thick catalyst layer (can reach 50 μm at 2 mg cm^–2^ for the Pt/C).^38^ Therefore, benefitting from the promoted formic acid oxidation and optimized mass transfer, the DFAFC with the PtCu NW GDE demonstrated excellent power performance compared to those reported in recent studies summarized in Table S3.

**Figure 6:**
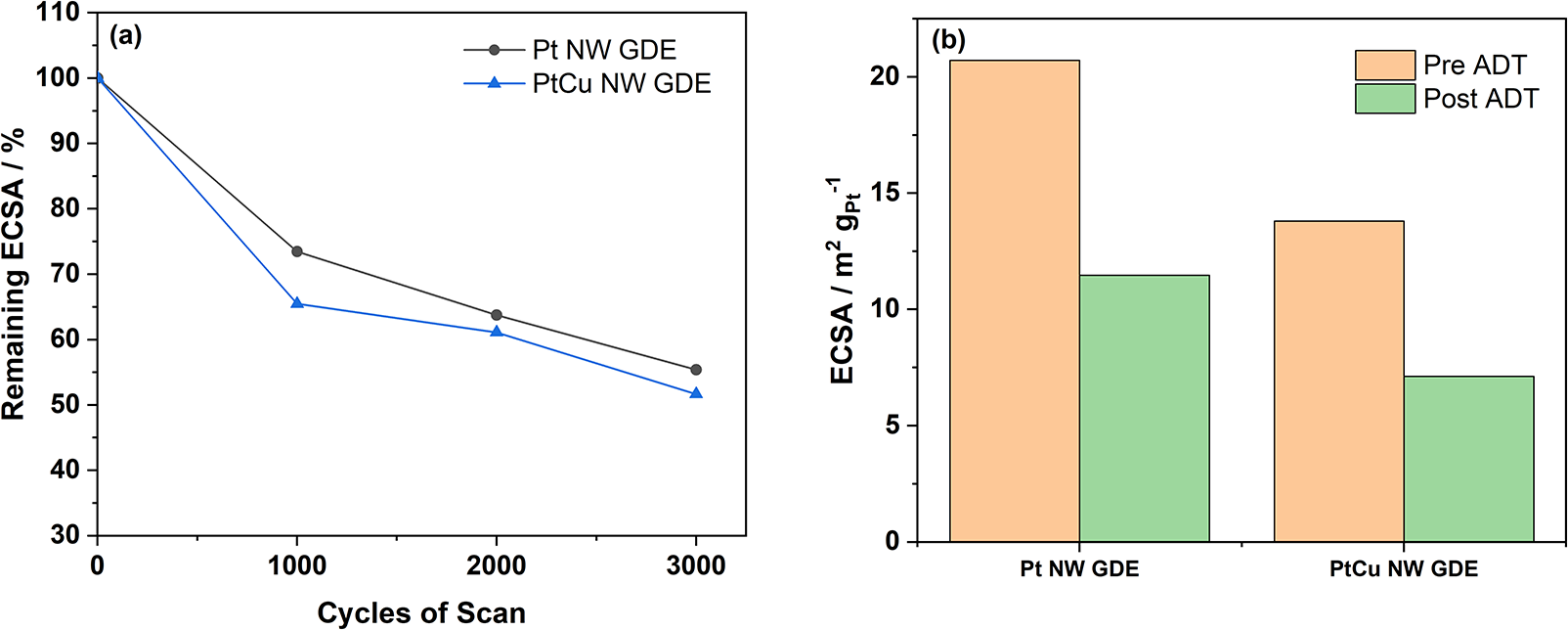
ADT for the Pt NW and Pt_3_Cu_1_ NW GDE in MEAs: (a) change of the ECSA with the potential scan cycle, and (b) comparison of the ECSA before and after the ADT.

1D Pt nanostructured catalysts have been widely reported to demonstrate excellent stability in the literature.^21^ To evaluate the durability of as-prepared PtCu NW GDE, ADT with the anode potential scan of 3000 cycles was performed on the MEAs made of the Pt NW GDE and Pt_3_Cu_1_ NW GDE, respectively. The CV changes are shown in Figure 6a, and the details are presented in Figure S7. With all MEAs, a significant ECSA drop occurs within the first 1000 cycles due to aggregation, dissolution, and Ostwald ripening. Within them, the PtCu NW GDE showed a slightly faster ECSA drop, which can be ascribed to the dissolution of Cu and the rearrangement of Pt atoms. After that, the downward trend flattens out, and PtCu NW GDE demonstrated a similar drop rate as that of the Pt NW GDE. Figure 6b compares their ECSA before and after the ADT. An ECSA loss of 44.9% is detected for the Pt NW GDE compared with 48.3% of the PtCu NW GDE. Compared with Pt nanoparticles reported in the other studies, these results reveal that the 1D nanostructure Pt and alloy have higher tolerance toward catalyst degradation, which is in line with our previous research.^24,39^

## 4 Conclusions

In this work, a new strategy is demonstrated to fabricate PtCu NW GDEs as the direct anode for DFAFCs. Arrays of PtCu alloy nanowires along the <111> direction are directly grown on the GDL surface using the formic acid reduction method and can be directly used as the gas diffusion electrode (GDE). The introduction of Cu atoms promotes the direct pathway mechanism of the formic acid oxidation due to the alloy effect, and the third-body effect that occurs with the addition of Cu atoms interrupts the arrangement of Pt atoms and curbs the formation of adsorbed CO intermediates. However, the alloying with Cu also results in the formation of large agglomerated clusters of nanowires and consequently lowers the ECSA and mass activity. During the fuel cell operation, the nanowire electrode structure significantly reduces the mass transport overpotential and leads to a much higher power performance. The Pt_3_Cu_1_ nanowire GDE shows the highest power density of 116.3 mW cm^–2^ due to the improved mass transport characteristics and CO intermediate tolerance, which is 2.4-fold higher in comparison to that of the Pt NW GDE.

## Supporting Information Available

Additional characterization results, including SEM, EDS mapping, TEM, ICP-MS, XPS spectra, ex situ GDE measurements, and single cell measurements and summary of DFAFC performance published in recent studies (PDF)
