# Supplementary material for: Catalyst Electrodes with PtCu Nanowire Arrays In Situ Grown on Gas Diffusion Layers for Direct Formic Acid Fuel Cells
Source: ACS Appl Mater Interfaces. 2022 Feb 24;14(9):11457–64. doi: 10.1021/acsami.1c24010 (PMC9007414; doi:10.1021/acsami.1c24010)
Supplement: Supplementary file 1 — am1c24010_si_001.pdf [file am1c24010_si_001.pdf]

Support information

Catalyst Electrodes with PtCu Nanowire Arrays in-situ Grown on Gas Diffusion Layers for Direct Formic Acid Fuel Cells

Yang Li,[a] Yichang Yan,[a] Yanping He,*[b] Shangfeng Du*[a]

[a]School of Chemical Engineering, University of Birmingham, Birmingham B15 2TT, UK

[b]School of Chemical Engineering, Kunming University of Science and Technology, Kunming 650504, China

*E-mail: YH, grace.he1985@hotmail.com; SD, s.du@bham.ac.uk

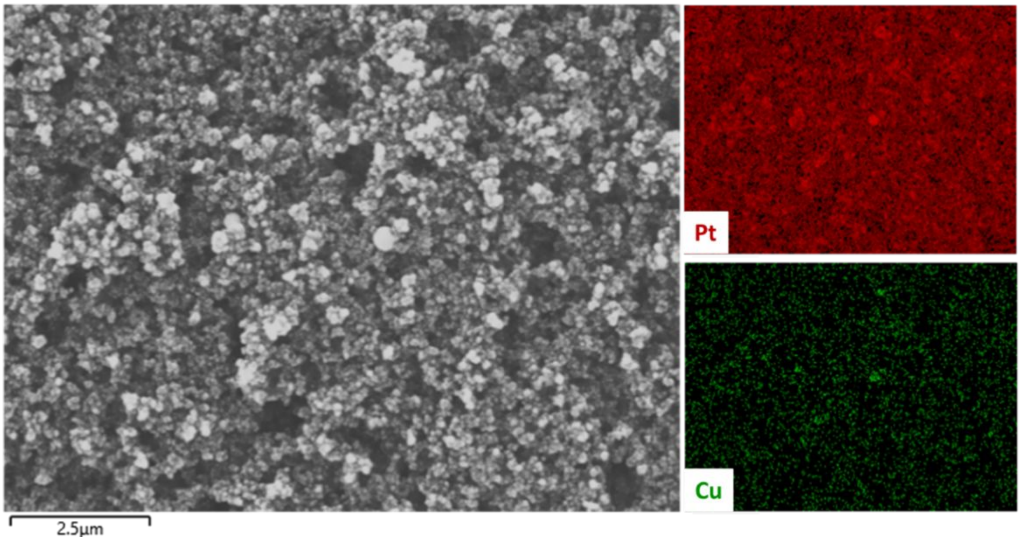

Figure S1 Surface SEM image and EDX mapping of the Pt_3Cu_1 NW GDE surface.

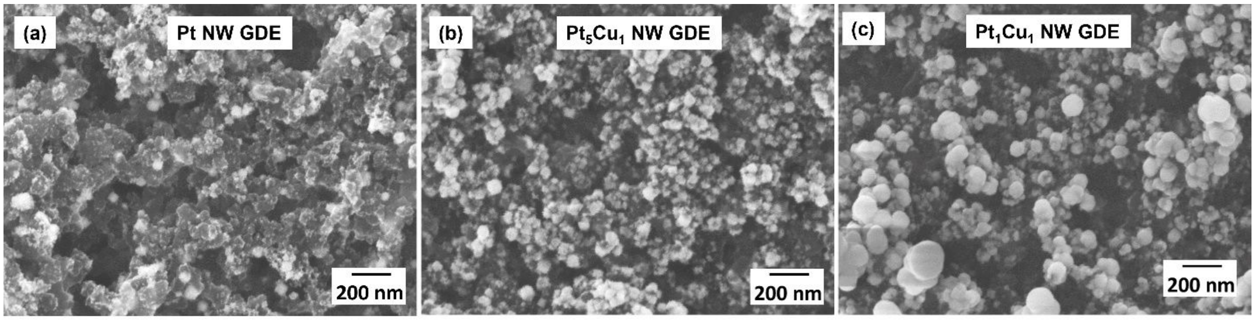

Figure S2 Surface SEM images of (a) Pt NW, (b) Pt_5Cu_1 NW and (d) Pt_1Cu_1 NW GDEs.

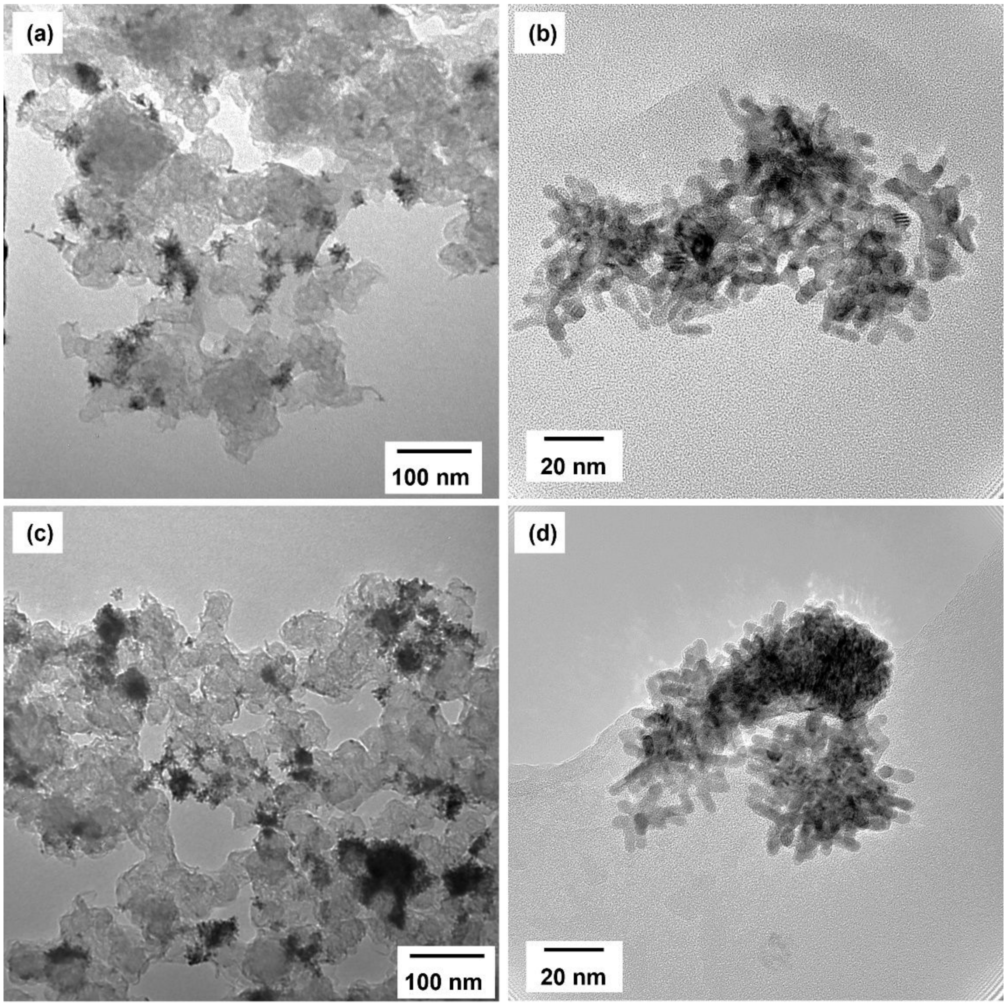

Figure S3 TEM images of typical clusters of (a, b) Pt and (c, d) Pt_3Cu_1 catalysts scraped from the GDE surface.

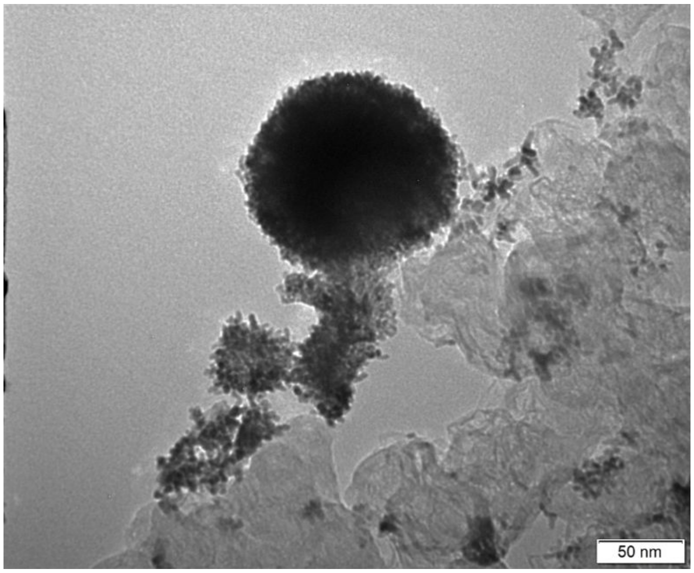

Figure S4 TEM image of typical clusters of Pt_1Cu_1 catalysts scraped from the GDE surface.

Table S1 The metal content of PtCu GDEs measured by ICP-MS analysis

Catalyst	ICP-MS / wt%		ICP-MS / at%	
	Pt	Cu	Pt	Cu
Pt_5Cu_1	92.73	7.27	80.59	19.41
Pt_3Cu_1	89.23	10.77	72.96	27.04
Pt_1Cu_1	75.19	24.81	49.68	50.32

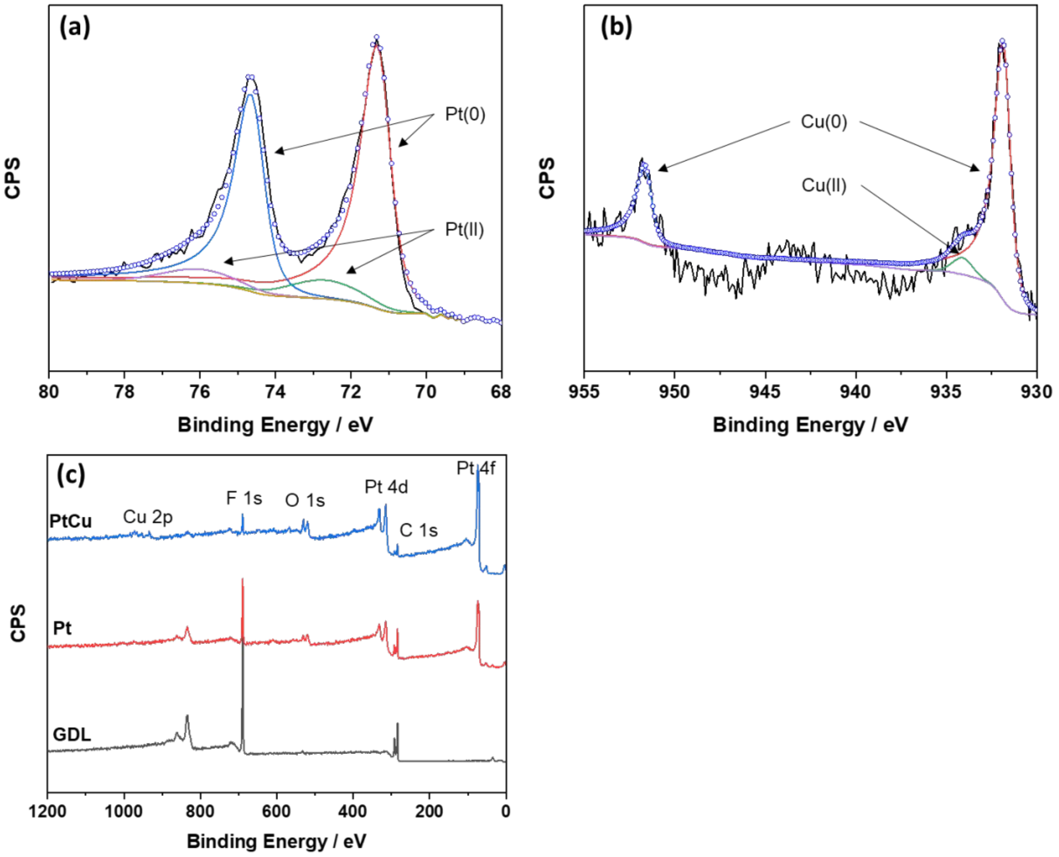

Figure S5 XPS patterns of high-resolution (a) Pt 4f and (b) Cu 2p of the PtCu catalysts on the GDL. (c) Comparison of XPS survey patterns of the PtCu GDE, Pt GDE and the GDL.

Table S2 Binding Energy and Pt(0) percentage deduced from the XPS spectra for the high-resolution Pt 4f region.

	**Binding Energy / eV**		**Percentage / %**
	**Pt 4f$_{7/2}$**	**Pt 4f$_{5/2}$**	**Pt(0)**
**Pt NW**	71.281	74.631	83.98
**Pt_5Cu_1 NW**	71.240	74.590	83.98
**Pt_3Cu_1 NW**	71.232	74.582	85.10
**Pt_1Cu_1 NW**	71.131	74.481	87.20

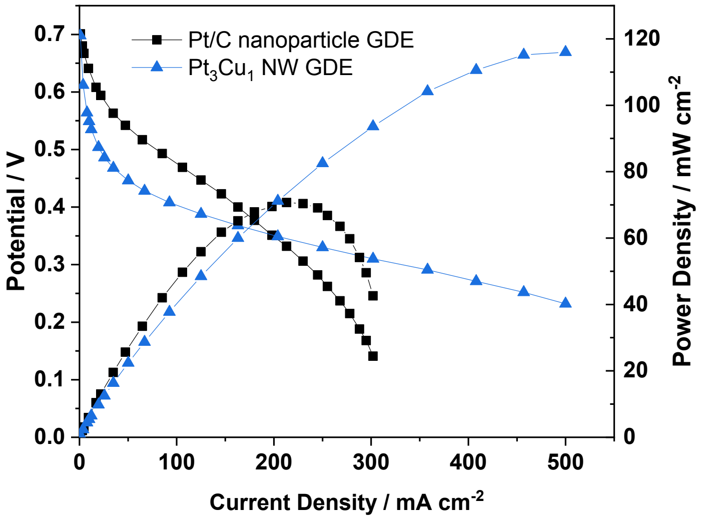

Figure S6 Polarization and power density curves of MEAs with anodes made of the Pt/C nanoparticle GDE and the Pt_3Cu_1 NW GDE.

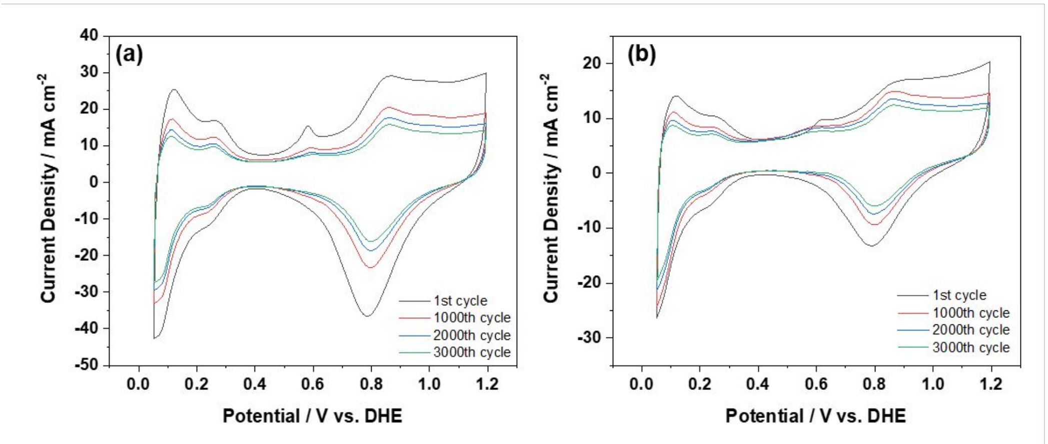

Figure S7 Anode CV curves during the ADT for MEAs made of (a) the Pt NW GDE and (b) Pt_3Cu_1 NW GDE.

Table S3 Comparison of the DFAFC performance reported in recent studies

Anode catalyst	Anode catalyst loading / mg cm^{-2}	Cathode catalyst	Cathode catalyst loading / mg cm^{-2}	Temp. / °C	Concentration of HCOOH	Flow rate of fuel / mL min^{-1}	Gas at cathode	Flow rate of gas / mL min^{-1}	Peak power density / mW cm^{-2}
PtCu (this work)	2.0	46%Pt/C	2.0	75	3M	1	Air	300	116
PdFe [1]	0.5	40%Pt/C	0.5	70	7M	1	O_2	200	160
PdFe [2]	1.2	40%Pt/C	0.8	65	3M	20	Air	1000	137
PtCu/carbon capsule [3]	2.4	60%Pt/C	2.4	80	3M	3	O_2	200	121
PtZn/carbon shell [4]	1.8	60%Pt/C	2.4	80	3M	3	O_2	200	107
Bi-Pt [5]	1.2	PtB	3.0	60	6M	5	Air	500	191
Bi-PtAu [6]	3.0	PtB	3.0	60	6M	5	Air	500	135
Pt [7]	2.0	Pt/C	2.0	70	3M	2	O_2	100	42
PdBi [8]	1.2	40%Pt/C	0.8	65	3M	10	Air	800	20
Pd/MWCNT [9]	0.5	Pt/C	1.0	60	3M	10	O_2	500	35
Pd-CoP [10]	0.3	PtB	4.0	30	3M	2	O_2	200	150

References

(1) Shan, J.; Lei, Z.; Wu, W.; Tan, Y.; Cheng, N.; Sun, X. Highly Active and Durable Ultrasmall Pd Nanocatalyst Encapsulated in Ultrathin Silica Layers by Selective Deposition for Formic Acid Oxidation. *ACS Appl. Mater. Interfaces* **2019**, *11* (46), 43130–43137. https://doi.org/10.1021/acsami.9b13451.

(2) Yang, S.; Chung, Y.; Lee, K.-S.; Kwon, Y. Enhancements in Catalytic Activity and Duration of PdFe Bimetallic Catalysts and Their Use in Direct Formic Acid Fuel Cells. *J. Ind. Eng. Chem.* **2020**, *90*, 351–357. https://doi.org/10.1016/j.jiec.2020.07.034.

(3) Xu, M.; Chen, H.; Zhao, Y.; Ni, W.; Liu, M.; Xue, Y.; Huo, S.; Wu, L.; Yang, Z.; Yan, Y. M. Ultrathin-Carbon-Layer-Protected PtCu Nanoparticles Encapsulated in Carbon Capsules: A Structure Engineering of the Anode Electrocatalyst for Direct Formic Acid Fuel Cells. *Part. Part. Syst. Charact.* **2019**, *36* (7), 1–10. https://doi.org/10.1002/ppsc.201900100.

(4) Xu, M.; Zhao, Y.; Chen, H.; Ni, W.; Liu, M.; Huo, S.; Wu, L.; Zang, X.; Yang, Z.; Yan, Y. M. Role of Ultrathin Carbon Shell in Enhancing the Performance of PtZn Intermetallic Nanoparticles as an Anode Electrocatalyst for Direct Formic Acid Fuel Cells. *ChemElectroChem* **2019**, *6* (8), 2316–2323. https://doi.org/10.1002/celc.201900332.

(5) Choi, M.; Ahn, C. Y.; Lee, H.; Kim, J. K.; Oh, S. H.; Hwang, W.; Yang, S.; Kim, J.; Kim, O. H.; Choi, I.; Sung, Y. E.; Cho, Y. H.; Rhee, C. K.; Shin, W. Bi-Modified Pt Supported on Carbon Black as Electro-Oxidation Catalyst for 300 W Formic Acid Fuel Cell Stack. *Appl. Catal. B Environ.* **2019**, *253* (September 2018), 187–195. https://doi.org/10.1016/j.apcatb.2019.04.059.

(6) Yoo, J. K.; Choi, M.; Yang, S.; Shong, B.; Chung, H.-S.; Sohn, Y.; Rhee, C. K. Formic

Acid Electrooxidation Activity of Pt and Pt/Au Catalysts: Effects of Surface Physical Properties and Irreversible Adsorption of Bi. *Electrochim. Acta* **2018**, *273*, 307–317. https://doi.org/10.1016/j.electacta.2018.04.071.

(7) Muthukumar, V.; Chetty, R. Morphological Transformation of Electrodeposited Pt and Its Electrocatalytic Activity towards Direct Formic Acid Fuel Cells. *J. Appl. Electrochem.* **2017**, *47* (6), 735–745. https://doi.org/10.1007/s10800-017-1076-z.

(8) Yang, S.; Yang, J.; Chung, Y.; Kwon, Y. Pd Bi Bimetallic Catalysts Including Polyvinylpyrrolidone Surfactant Inducing Excellent Formic Acid Oxidation Reaction and Direct Formic Acid Fuel Cell Performance. *Int. J. Hydrogen Energy* **2017**, *42* (27), 17211–17220. https://doi.org/10.1016/j.ijhydene.2017.06.018.

(9) Zhang, W.; Yao, Q.; Wu, X.; Fu, Y.; Deng, K.; Wang, X. Intimately Coupled Hybrid of Graphitic Carbon Nitride Nanoflakelets with Reduced Graphene Oxide for Supporting Pd Nanoparticles: A Stable Nanocatalyst with High Catalytic Activity towards Formic Acid and Methanol Electrooxidation. *Electrochim. Acta* **2016**, *200*, 131–141. https://doi.org/10.1016/j.electacta.2016.03.169.

(10) Feng, L.; Chang, J.; Jiang, K.; Xue, H.; Liu, C.; Cai, W.-B.; Xing, W.; Zhang, J. Nanostructured Palladium Catalyst Poisoning Depressed by Cobalt Phosphide in the Electro-Oxidation of Formic Acid for Fuel Cells. *Nano Energy* **2016**, *30*, 355–361. https://doi.org/10.1016/j.nanoen.2016.10.023.
